# Correlation between preoperative predictions and surgical findings in the parotid surgery for tumors

**DOI:** 10.1186/s13005-016-0100-6

**Published:** 2016-01-12

**Authors:** Michael Vaiman, Judith Luckman, Tal Sigal, Inessa Bekerman

**Affiliations:** Department of Otolaryngology – Head and Neck Surgery, Assaf Harofe Medical Center, Affiliated to Sackler Faculty of Medicine, Tel Aviv University, Zerifin, Israel; Department of Radiology, Neuroradiology section, Beilinson campus, Rabin medical center, Holon, Israel; Department of Radiology, Assaf Harofe Medical Center, Affiliated to Sackler Faculty of Medicine, Tel Aviv University, Zerifin, Israel

**Keywords:** Parotidectomy, Parotid tumors, CT, MRI, Deep lobe parotidectomy

## Abstract

**Background:**

To compare preoperative CT/MRI based predictions with real surgical findings for deep lobe parotid gland surgery.

**Methods:**

The study analyzed 122 parotidectomies (2004–2014) for benign tumor removal. The facial nerve, the Utrecht line, the Conn’s arc, and the retromandibular vein were used as landmarks for CT/MRI presurgical evaluation of patients. We assessed 106 CT images and 86 MRI images. The study compared preoperative evaluation of tumor location with its actual location that was revealed during the operation and assessed the importance of the landmarks.

**Results:**

In general, the agreement between preoperative CT prediction and actual location of the parotid tumors was achieved in 88.7 % (*n* = 94/106) when facial nerve line was used as a landmark. However, out of 14 tumors in the deep lobe only 5 were located correctly (35.7 %). Of the other existing CT landmarks, none showed more precision over others. The agreement between MRI based prediction and surgical results on actual location of the tumor was achieved in 94.2 %. Out of 12 MRI-investigated tumors in the deep lobe nine were located correctly that gives 75 % agreement with surgical results.

**Conclusion:**

Our data suggests that no existing CT landmark can be accepted as completely reliable in cases when selective deep lobe parotidectomy is planned. If tumor location is suspected in the deep lobe of the gland, MRI imaging is necessary to confirm the diagnosis. An operating surgeon should be prepared that in some cases the true location of the tumor would be revealed only during surgery.

## Background

The precise identification of location of benign tumors of the parotid gland in the superficial or deep lobes can help to avoid total parotidectomy. In addition to gland preservation, in cases when only the deep lobe is affected selective parotidectomy can help to preserve the facial nerve, avoid Frey’s syndrome (gustatory sweating), and provide better aesthetic/cosmetic results.

**Fig. 1:**
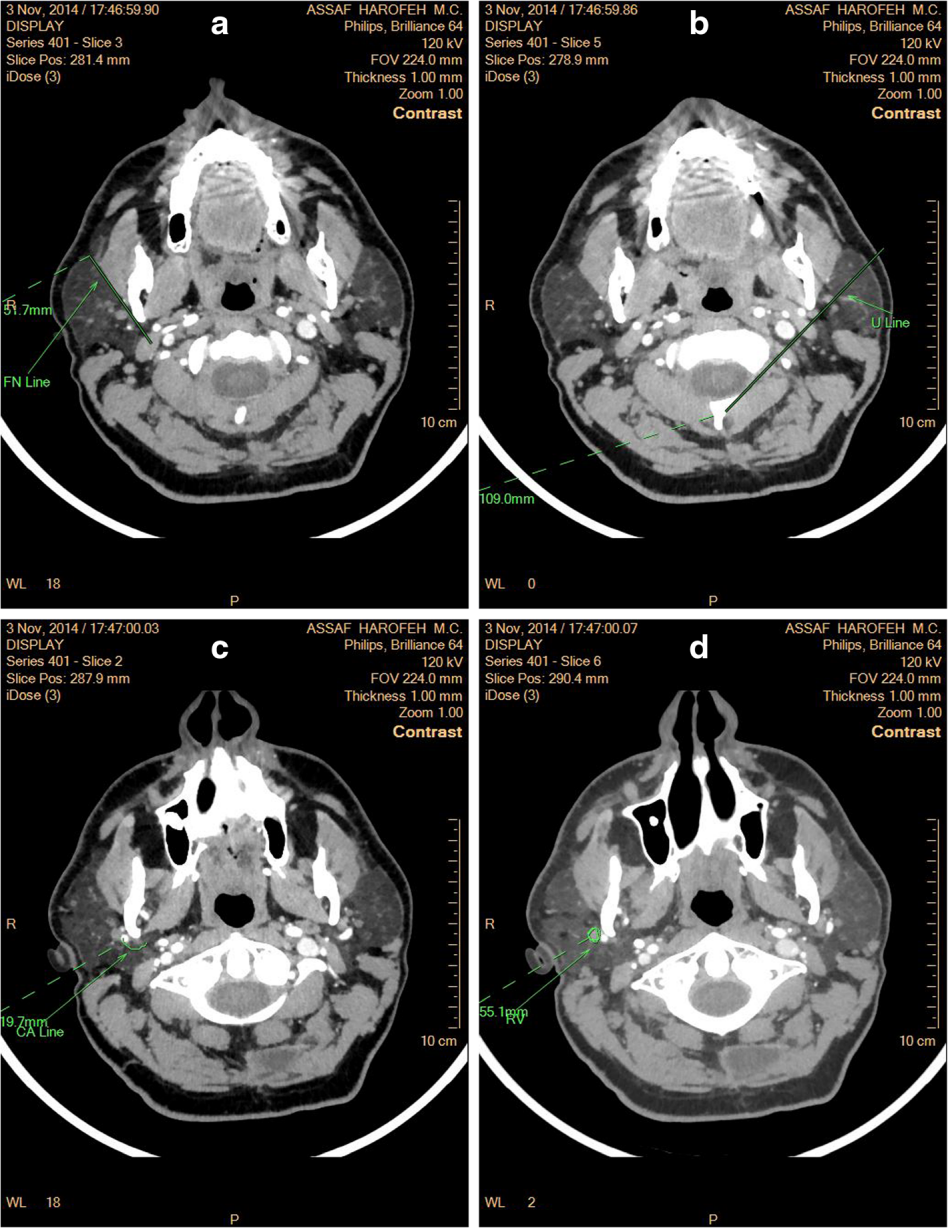
**a** The facial nerve line (FNL) is the line between the lateral surface of the posterior belly of *m. digastricus* and the lateral surface of the cortex of the ramus part of the mandible as seen at CT scan image. **b** The Utrecht line (UL) runs from the most dorsal point of the ipsilateral half of the first vertebra to the most dorsal point of the retromandibular vein as seen at CT scan image. **c** The Conn’s arc (CA) is a 8.5 mm radius semicircle with the center on the most distant point of the posterior edge of the ramus as seen at CT scan image. **d** The retromandibular vein line (RV) as seen at CT scan image

Various landmarks were used in computed tomography (CT) and magnetic resonance imaging (MRI) investigations for precise localization of the tumor and of the facial nerve. Among these landmarks, the facial nerve line (FNL) is the line between the lateral surface of the posterior belly of *m. digastricus* and the lateral surface of the cortex of the ramus part of the mandible [1, 2]. The Utrecht line (UL) runs from the most dorsal point of the ipsilateral half of the first vertebra to the most dorsal point of the retromandibular vein [3, 4]. The Conn’s arc (CA) is a 8.5 mm radius semicircle with the center on the most distant point of the posterior edge of the ramus [1, 5]. In addition to these lines, the retromandibular vein (RV), the styloid process, the lateral border of the masseter, the lateral border of the mandible, and the Stensen’s duct were used as landmarks also [3, 6, 7]. Definitely some disagreement exists on the question which landmark or line is the most reliable for preoperative diagnostics. While some authors name FNL as the most reliable landmark [2, 3], the other suggest UL [1] or the Stensen’s duct [6]. These landmarks are presented at the Fig. 1a-d.

To answer this question, we compared preoperative CT/MRI based predictions with real surgical findings for parotid gland surgery.

## Methods

### Study design and setting

The retrospective study analyzed 122 surgical operations (2004–2014) for different types of partial parotidectomy. The study compared preoperative evaluation of tumor location with its actual location that was revealed during the operation and assessed the importance of the landmarks. The study protocol conformed to the ethical guidelines of the 1975 Declaration of Helsinki (amended 2000) as reflected a priori after approval by the institution’s Helsinki committee.

The *inclusion criteria* were as follows. All selected patients had primary benign parotid tumors. From 2004 to 2014, 142 parotidectomies were performed to remove benign tumors. Of them, CT or CT + MRI were performed for 122 patients and in 20 cases clinical picture, ultrasonography and FNAB was enough to confirm the diagnosis. These 122 cases were selected for analysis. We analysed data that was obtained by the operating surgeons before the surgery and compared preoperative CT/MRI based predictions with real surgical findings for parotid gland surgery. The cases with malignant tumors were excluded from the analysis because total parotidectomies were performed disregarding the location of the tumor.

### Data sources and measurements

All the CT scans were obtained by the 256-slice CT scanner (Brilliance iCT, Philips Healthcare, The Netherlands) with NanoPanel 3D spherical detectors in axial (transverse) plane that were used further for reconstruction of coronal (frontal) and sagittal planes (spine window, middle third; window parameters: WW 60, WL 360, accuracy 1 pixel). The standard Philips protocols for head and neck imaging were implemented in all cases with slices performed at 25° to the skull base. When the CT scans were obtained, the parotid glands were analyzed using FNL, UL, CA, and RV landmarks. All measurements were made using the same window, contrast and brightness.

The MRI parameters were as follows: Precontrast: a coronal TIRM sequence: TR, 5580 ms; TE, 61 ms; section thickness, 3 mm; FOV, 287 mm; resolution, 624; an axial T2 fs-dixon sequence: TR, 4010 ms; TE, 79 ms; section thickness, 3 mm; FOV, 280 mm; resolution, 739; an axial T1-weighted fs-dixon TSE sequence: TR, 590 ms; TE, 11 ms; section thickness, 3 mm; FOV, 586 mm; resolution, 1215; a coronal T1-weighted fs-dixon TSE sequence: TR, 670 ms; TE, 10 ms; section thickness, 3 mm; FOV, 548 mm; resolution, 1132. Post contrast: an axial gadolinium-enhanced T1 VIBE fat-saturated sequence 1 + 4; TR, 3.78 ms; TE, 1.25 ms; section thickness, 4 mm; FOV, 192 mm; resolution, 439; an axial T1 fs-dixon TSE fat-saturated sequence; a coronal gadolinium-enhanced T1 TSE fat-saturated sequence: TR, 613 ms; TE, 12 ms; section thickness, 3 mm; FOV 410 mm; resolution, 871 and a sagittal gadolinium-enhanced T1 TSE fat-saturated sequence: TR, 628 ms; TE, 8.7 ms; section thickness, 3 mm; FOV, 400 mm; resolution, 868.

While CT scans were performed in 106 cases, the MRI was performed in 86 cases for further confirmation of the diagnosis. Of them, both CT and MRI were performed in 70 cases and in 16 cases only MRI was performed.

### Analysis

The error margin was expressed by means of the technical error of measurement (TEM) to calculate the inter-evaluator variability between two initial evaluators (authors 3 and 4 of the submission). The same equipment and methodological procedures for measurements were adopted by both evaluators. When the initial results were obtained, an independent evaluator was invited from another institution to re-evaluate the results (author 2 of the submission).

The questions put to be answered by the CT/MRI investigation were as follows: 1) the location of the tumor (deep lobe/superficial lobe), 2) encapsulation of the tumor (yes/no), 3) tumor’s progression into the parapharyngeal space (yes/no). The data were statistically evaluated by three-dimensional analysis of variance, SPSS, Standard version 17.0 (SPSS, Chicago, IL, 2007), and χ^2^ criterion using 95 % confidence interval. The level of significance for all analyses was set at *p* < 0.05.

## Results

From 2004 to 2014, 450 patients underwent CT/MRI of the parotid region. Of them, 106 patients were diagnosed with benign neoplasms of the parotid gland and 11 were diagnosed with malignant neoplasms. In addition to CT, MRI, or CT/MRI investigations performed for 122 cases selected for analysis, the diagnosis was confirmed by ultrasonography and FNAB for all these patients. Among these selected cases, there were 58 (47.55 %) females and 64 (52.45 %) males with a mean age of 43 years (18–80 years) in the analyzed cohort of benign tumors. Three different surgeons experienced in salivary gland surgery operated on these patients. The patients selected for surgery were diagnosed with pleomorphic adenoma (*n* = 60; 49.2 %), Warthin’s tumor (*n* = 32; 26.2 %), lipoma (*n* = 26; 21.3 %), and hemangioma (*n* = 4; 3.3 %). Successful parotidectomy was achieved in 97.5 % (*n* = 119) of the operated cases. In three cases reoperation with gland excision was needed.

Based on ultrasonography, CT, or MRI data, the type of the surgery was chosen. All surgeries were performed under general anaesthesia. In all cases, after-surgery follow-up was scheduled at 1, 3, 6, 12, and 24 months after the procedure, however only 64 patients were under follow-up for 12 month and only 33 appeared after 24 month after the surgery. The six-month follow-up revealed an absence of symptoms in all cases. Recovery of three patients with gland excision was successful.

### Comparison between CT/MRI findings and the surgery results

For inter-evaluator TEM, difference between evaluators varied from 3.38 to 3.75 for different questions (acceptable). For TEM between the initial conclusions and the independent evaluator re-evaluation difference varied from 3.5 to 4.2 (acceptable).

By preoperative CT prediction with the help of FNL landmark, eight of the patients had the tumor located in the deep lobe of the gland and in 98 cases the lesion was diagnosed in the superficial lobe. As it was found during surgery, out of 106 cases 92 tumors were located in the superficial lobe of the gland and 14 tumors appeared to be in the deep lobe. In addition to this, out of eight cases initially diagnosed in the deep lobe only five were found there and another three were found in the superficial lobe. Therefore, general agreement between preoperative prediction and actual location of the superficial or deep lobe tumors was achieved in 88.7 % (12 mistakes out of 106 cases). However, out of 14 tumors in the deep lobe only five were located correctly (35.7 %).

The other existing landmarks were less precise:

Utrecht line (UL) vs. surgery 83 % (18 mistakes/106 cases);

The Conn’s arc (CA) vs. surgery 71.7 % (30 mistakes/106 cases);

Retromandibular vein (RV) vs. surgery 73.6 % (28 mistakes/106 cases);

The main body of mistakes concerned cases with tumors in the deep lobe. Data on specificity and sensitivity of the above mentioned CT landmarks are presented in Table 1.

**Table 1:** Specificity and sensitivity of CT investigation for tumors of the parotid gland (*n* = 106). The variables investigated: (1) *tumor location in the superficial lobe (yes/no),* (2) *tumor location in the deep lobe (yes/no),* (3) *encapsulation of the tumor (yes/no),* and (4) *progression into the parapharyngeal space (yes/no)*

Variable	TP	FP	TN	FN	specificity	sensitivity
Location: superficial lobe						
Facial nerve line	87	3	5	11	0.88	0.94
Utrecht line	56	7	7	36	0.5	0.6
The Conn’s arc	55	8	6	37	0.57	0.59
Retromandibular vein	64	7	7	28	0.5	0.69
Location: deep lobe						
Facial nerve line	5	3	89	9	0.84	0.36
Utrecht line	6	8	84	8	0.79	0.43
The Conn’s arc	7	5	87	7	0.82	0.5
Retromandibular vein	9	10	82	5	0.77	0.64
Encapsulation	101	0	4	1	0.95	0.99
parapharyngeal space	4	2	97	3	0.8	0.95

*Abbreviations*: *TP* true positive, *FP* false positive, *TN* true negative, *FN* false negative

Analysis of MRI imaging presented excellent results in detecting encapsulation of the tumor and tumor’s progression into the parapharyngeal space (100 % agreement with surgical results). Agreement between preoperative prediction and actual location of the tumor was achieved in 94.2 % (5 mistakes/86 cases). The four mistakes however were made in deep lobe cases. Out of 12 MRI-investigated tumors in the deep lobe nine were located correctly that gives 75 % agreement with surgical results. Data on specificity and sensitivity of MRI imaging analysis are presented in Table 2.

**Table 2:** Specificity and sensitivity of MRI investigation for tumors of the parotid gland. Tumor location was assessed using the facial nerve line as the most sensitive

Variable	specificity	sensitivity
Tumor location in the superficial lobe (yes/no)	0.95	0.95
Tumor location in the deep lobe (yes/no)	1	0.8
Encapsulation of the tumor (yes/no)	1	1
progression into the parapharyngeal space (yes/no)	1	1

## Discussion

The location of the tumor proportional to the retromandibular vein, the styloid process, the lateral border of the masseter, the lateral border of the mandible, and the Stensen’s duct was detected and the relation to the facial nerve was analyzed by the surgeons before the operation and further clarified during the operation. Analyzing the obtained data we can see that while both CT and MRI are very sensitive and specific in questions of tumor encapsulation and progression to the spaces out from the parotid gland itself, the abilities of these investigative methods to localize a lesion within the glandular tissue are less impressive, especially for CT.

One may say that FNL landmark for CT images was satisfactory precise by achieving 88.7 % agreement with surgical data. However, its precision for deep lobe location was only 35.7 % that is not satisfactory at all. Other landmarks showed worse agreement. It means that if a surgeon suspects a tumor in the deep lobe and plans selective deep lobe parotidectomy with superficial lobe preservation, he/she cannot completely rely on obtained CT imaging and is forced to add MRI data.

Some previous publications indicate high sensitivity and specificity (up to 100 %!) of CT based preoperative location of the parotid tumor [1, 2, 8]. We understand it as a wrong interpretation of statistical reports. Finding general sensitivity/specificity for cases of the tumor has sense if tumor distribution is equal in all parts of a gland or any other organ. In case of the parotid gland, most of the cases of tumor, up to 81-90 %, are found in the superficial lobe [9–13]. Suppose, a researcher had 100 cases of a tumor, of them 90 cases were located in the superficial lobe and 10 were located in the deep lobe. Suppose, a radiologist indicated all 100 cases to be located in the superficial lobe and is satisfied with 90 % agreement, but for a surgeon who could perform selective deep lobe parotidectomy instead of total or superficial parotidectomy this would be a total failure. This particular surgeon will not welcome such “90 % agreement” with any visible outburst of joy.

Precise localization of the tumor is very important. Rarely today but still parotidectomy may cause permanent damage to nerve such as facial paralysis and Frey’s syndrome after surgery due to the anatomical interralations between the parotid gland and the facial nerve (FN). Currently, superficial parotidectomy causes minimal risk to the FN in most of the cases, whereas surgery for tumors in the deep lobe of the gland has a higher rate of FN injury. If total parotidectomy is necessary, however, the FN would be injured because of exposing FNs even when they are not sacrificed. Therefore, accurate preoperative evaluation of the location of the parotid gland tumor is important for the surgical outcomes and prognosis of patients because its location significantly affects the time and difficulty of operation.

As it was said above, various researchers commented on many predicting methods, such as CA, FN line, U line, and RV, to identify the location of the parotid gland tumor before operation using CT imaging and predicted the relationship between the location of the tumor and these landmarks. Satisfactory results were reported but in practice the idea did not work out quite so well. That is why we agree with those authors who stated that MRI is dominant for determining tumor location and facial nerve involvement [14]. When identifying tumor location using CT or MRI imaging, the FN branches in the CT image cannot be detected even with contrast media. It is common sense that MRI is more sensitive than CT but we inclined to agree with those authors who indicated that even MRI cannot achieve 100 % correct diagnosis [3, 15].

Recently, another approach to the problem was introduced suggesting the suprahyoid neck to be divided into characteristic anatomic spaces, which allow for the accurate localization of both normal and abnormal elements in the neck [16]. While dealing mostly with sublingual and submandibular glands, this method might be extrapolated to the parotid region and perhaps practitioners and radiologists will obtain more precise method for localization of deep lobe lesions.

### Limitations of the research

All the CT scans and MRI images were obtained as described above. It might be possible that scanners of different trademarks could provide slightly different results of measurements as well as sonography evaluation. We evaluated benign tumors of the gland and different approach might be applied to malignant tumors.

### Generalisability

External validity of the study results is based on recent efforts in standardization of CT and MRI nomenclature and protocols for various scanner manufacturers (GE, Philips, Toshiba, Hitachi, Siemens). All these manufacturers provide features to automatically initiate a prescribed axial, helical or dynamic scan when a threshold level of contrast enhancement is reached at a specified region of interest (in our case, the parotid gland) [17].

## Conclusion

Our data suggests that no existing CT landmark can be accepted as completely reliable in cases when selective deep lobe parotidectomy is planned. If tumor location is suspected in the deep lobe of the gland, MRI imaging is necessary to confirm the diagnosis. An operating surgeon should be prepared that in some cases the true location of the tumor would be revealed only during surgery.
